# Response to “Older Cancer Patients’ User Experiences With Web-Based Health Information Tools: A Think-Aloud Study”

**DOI:** 10.2196/jmir.6613

**Published:** 2016-11-16

**Authors:** Shyam Ajay Gokani, Georgina Kerry, Ekta Sharma, Javier Ash, Alexander Kamiar Zargaran, Dara Shalini Rasasingam, Aaina Mittal

**Affiliations:** ^1^ Imperial College Business School Imperial College London London United Kingdom; ^2^ Faculty of Life Sciences and Medicine King’s College London London United Kingdom

## Letter

We read with great interest the paper by Bolle et al that highlights the important determinants of usability and perceived usefulness of Web-based health information among older adults [1]. This paper is both timely and important. Web-based health information is increasingly being seen as an efficient means of patient education; however, the health information needs of older adults are often neglected [2]. Therefore, understanding how to improve Web-based tools for older adults would be hugely beneficial.

The authors conclude that older cancer patients are able to use cancer information websites and find them useful. However, there are certain clarifications that seem necessary before adopting these findings into common practice.

Firstly, we note that participants were recruited from PanelCom, a service which recruits cancer patients via email and conducts most studies online [3]. Such patients are likely to be more experienced with Web-based technology than the average older adult and hence have a higher threshold for detecting usability issues.

Second, when searching for health information, patients tend to first use generic queries in Web search engines as opposed to directly accessing specific medical websites [4,5]. Therefore, the authors’ assessment of the navigational usability of these websites does not accurately reflect the true usage pattern of such websites.

Finally, it is important to bear in mind that Web-based tools must balance the differing needs of distinct patient groups. For example, increasing the size of text also increases the need for scrolling on a page, an issue which 9% of participants objected to. Hence recommendations that enhance the ability to personalize Web-based tools are preferred over generalized recommendations.

Notwithstanding these considerations, it is clear that factors such as the content delivered, readability, and the use of multimedia all influence the likelihood of the use of Web-based information tools by older adults. Other factors that might determine perceived usefulness also include the currency, authorship, and bias contained within health care information, all of which could be potential avenues for future research. Furthermore, although the authors touch upon the importance of interpersonal communication with physicians together with Web-based tools, the true value of integrating the tools within the patient consultation could also be further explored. Health care professionals are well placed to point patients using the Internet in the right direction and help them to identify relevant data amid an “information overload.” There is also evidence to suggest that the recommendation of Web-based tools by a physician increases perceived usefulness and compliance [6,7].

In light of these findings, there is still a need for good quality Web-based health information that considers the requirements of older adults. This study is valuable to help elucidate the path to developing useful informational tools for such a group of patients. Although some areas of clarification exist, the authors clearly make a unique contribution to a field in which there is a dearth of existing literature. Future designers of Web-based information tools would do well in considering the pertinent factors identified, in addition to others that have not been completely explored in the past.
